# Editorial: Health-related quality of life in health care

**DOI:** 10.3389/fpubh.2023.1123180

**Published:** 2023-02-02

**Authors:** Samer A. Kharroubi, Iffat Elbarazi

**Affiliations:** ^1^Department of Nutrition and Food Sciences, Faculty of Agricultural and Food Sciences, American University of Beirut, Beirut, Lebanon; ^2^School of Health and Related Research, The University of Sheffield, Sheffield, United Kingdom; ^3^College of Medicine and Health Sciences, Institute of Public Health, United Arab Emirates University, Al Ain, United Arab Emirates

**Keywords:** Health Related Quality of Life (HRQoL), health and patients outcomes, cost-effectiveness analyses (CEAs), patient-oriented research (POR), healthcare management, health policy, quality-adjusted life years (QALYs), decision making

Health Related Quality of Life (HRQoL) is a cornerstone in the evaluation of modern medicines, healthcare practice and other medical interventions (1). It is of increasing importance in healthcare in the context of the rapidly growing power, variety and expense of modern medicines. Whether generic or condition specific, many instruments have been developed to ensure that clinical studies consider the needs of patients (2–7). These can be used to demonstrate the efficacy of a new technology, to document the burden associated to a given disease and either to support or to inform health policy, pricing and reimbursement decision making (8).

In health economics, it is useful to perform cost-effectiveness and cost-utility studies by considering the preferences of patients (8, 9). The World Health Organization (WHO), for instance, undertakes cost-effectiveness and cost-utility assessments of interventions across national boundaries. In the UK, for example, the National Institute for Health and Clinical Excellence (NICE) determines which treatments and/or procedures should be used in the National Health Service (NHS) on the basis of their cost-effectiveness, and there are similar advisory or regulatory bodies in several other countries, including US, Canada, Australia and the Netherlands.

To date, plenty of research is conducted in this field and relates, for example, to the choice of the instrument, methodological development, ability to capture specific health dimensions, and lots more. Our Research Topic was open to the subject area of health-related quality of life in healthcare and in clinical practice. Articles included instrument development, new technologies, application, evaluations and outcomes. For example, Li et al. have evaluated the global, sex, age, region, and country-specific cardiovascular disease (CVD) burden, along with the trends, risk factors, and implications for the prevention of CVD. This study would help explain the characteristics of the global CVD burden in order to set up more effective and targeted prevention strategies. Lobo et al. have studied the need to address potential caregiver-affecting issues at many stages of health planning, recovery, and policymaking in order to generate the evidence to enable stroke caring engagement. In addition, a thorough understanding of the processes involved in stroke care, available services and individual needs and experiences to develop a more realistic approach toward engagement with whom. Cheng and Jin have investigated the association between smoking and HRQoL among Chinese individuals aged 40 years and older using an instrumental variable probit model. Their findings showed that smoking leads to a smaller probability of having a better quality of life, thereby anti-smoking campaigns are then needed to highlight the negative effect of tobacco use on HRQoL. In a multinational study, Ghazy et al. have also assessed the quality of life and its determinants among healthcare workers living and working in the Arab world. The findings of this study indicate that more attention should be administered to this key group in order to ensure their productivity and service provision. Besides, AlKuwaiti et al. have also explored the physical, social, psychological, spiritual, and lifestyle impact of a positive COVID-19 diagnosis on a sample of the UAE population. The study shows that patients with COVID-19 have perceived very good support in terms of their physical health from the government and health authorities, but need social, psychological, and educational support during the infection period and after immediate recovery and post-recovery.

With regards to cost-effectiveness analyses, Shi et al. have assessed the cost-effectiveness of the same-day discharge (SDD) in comparison to that of regular care for primary total hip arthroplasty patients. This is achieved by analyzing the effect using the Oxford hip score (OHS), medical costs (both out-of-pocket and reimbursed), mean incremental cost-effectiveness ratio (ICER), and quality-adjusted life years (QALYs) at 6-month follow up. By a standard cost-effectiveness analysis (CEA), this study would help physicians, government and health authorities to look at SDD in a more precise and efficient approach. Additionally, Shao et al. have also evaluated the cost-effectiveness of camrelizumab plus chemotherapy to treat sq-NSCLC from the Chinese healthcare system perspective. The findings of this economic evaluation approach would provide evidence for clinicians, government and health authorities in making optimal decisions in general clinical practice.

To this end, our Research Topic was sealed with a methodological paper where Kharroubi and Kelleher have developed a novel Bayesian non-parametric model for estimating the utility values of health states defined by a generic descriptive system, to generate quality-adjusted life years and hence to conduct cost utility analysis of healthcare interventions. The new Bayesian statistical model also permits the utilization of evidence from one country as potential prior information for a study in another. The results of this study suggest that existing countries' valuations could be used as informative priors to generate better utility estimates than modeling the data from each country separately. This sort of analysis (borrowing strength from existing countries) could be particularly promising in terms of reducing the need for conducting large surveys in every country, which would in turn reduce the cost of cross-country valuation. This will be hugely important for countries where large-scale evaluation exercises are expensive and hard to conduct, particularly for countries with small population size or low- and middle-income countries.

So as a final word, Health Related Quality of Life in Healthcare has become the focus of health related research and practice especially after the COVID-19 crisis which have unveiled systems weaknesses and the need for future preparedness with more evidence based oriented actions and polices which this topic has aimed for.

## Author contributions

SK participated in the conceptualization of the idea, the design of the Research Topic, editorial drafting, and the final review of the editorial. IB reviewed and finalized the editorial and participated in the conceptualization of the study. All authors have read and approved the final editorial.
